# Supplementary material for: Birds land reliably on complex surfaces by adapting their foot-surface interactions upon contact
Source: eLife. 2019 Aug 6;8:e46415. doi: 10.7554/eLife.46415 (PMC6684272; doi:10.7554/eLife.46415)
Supplement: Supplementary file 1. — (A) Surface roughness parameters. Roughness parameters are based on processing 3D scans of each surface (see Materials and methods). (B) Individual foot preferences and adjustments during landing. Just as humans exhibit hand preference, birds appear to employ a dominant foot when carrying out different tasks. A previous study considered foot dominance in the context of manipulating food and found that approximately 50% of the parrots studied were left-footed, 25% were right-footed, and the remaining were ambidextrous (Magat and Brown, 2009). In our study, based on which foot contacted the perch first, we found that one bird was right footed (100% of flights) while the other two were left footed (83% and 85% of flights). We also looked at how the birds adjusted their feet after making initial contact. The first foot to move was the dominant foot for two individuals, but not for the third. In addition, we found that the first foot adjustment was primarily in the forward direction (92%, 83%, and 72%). (C) Parrotlet foot adjustments on each perch. Parrotlets made, on average, about two adjustments after landing. The birds adjusted their feet the most on teflon (2.78 adjustments), and the least on the 0.25" diameter birch dowel (1.33 adjustments). (D) Foot kinematics parameters. The average foot spread angle while in the resting stage was approximately 36°. When approaching the perch, the birds began to open their feet approximately 90 ms before making contact with the surface. During this time, the foot angle reached a maximum of 172° on average, and this peak typically occurred approximately 31 ms before making contact with the surface. While pre-shaping, the foot angle decreased approximately 24° on average. Before making contact with the perch, the average claw angle was 177°. After having made contact, while wrapping the perch, the foot angle dropped another 30° on average. The claw angle, on the other hand, dropped an average of 42° during this stage. The claw reac [file elife-46415-supp1.docx]

Supplementary Tables for

**Birds land reliably on complex surfaces by adapting their foot-surface interactions upon contact**

**The PDF file includes:**

- Supplementary file 1A. Surface roughness parameters and hardness estimates.
- Supplementary file 1B. Individual foot preferences and adjustments during landing.
- Supplementary file 1C. Parrotlet foot adjustments on each perch.
- Supplementary file 1D. Foot kinematics parameters

|  | Arithmetical Mean Deviation (μm) | Root Mean Squared (RMS) Roughness (μm) | Peak Height (μm) | Peak Depth (μm) | Skewness |
| --- | --- | --- | --- | --- | --- |
| Birch | 9.26 | 12.04 | 55.59 | -91.67 | -0.4821 |
| CLO | 16.08 | 22.30 | 167.60 | -153.23 | 0.7413 |
| FS | 12.69 | 16.41 | 69.32 | -82.85 | -0.1825 |
| Foam | 9.39 | 13.72 | 150.31 | -177.98 | -2.2976 |
| SO | 40.71 | 50.87 | 170.87 | -181.91 | -0.3345 |
| Sandpaper | 67.07 | 84.89 | 508.16 | -336.87 | 0.2460 |
| Teflon | 3.92 | 4.99 | 55.83 | -22.77 | 0.1756 |

**Supplementary file 1A. Surface roughness parameters.**

|  | Parrotlet 1 | Parrotlet 2 | Parrotlet 3 |
| --- | --- | --- | --- |
| % of landings in which the bird’s right foot contacted the perch first | 100% | 17% | 15% |
| % of landings in which the bird’s first foot adjustment was with its right foot | 81% | 17% | 64% |
| % of landings in which the bird’s first foot adjustment was forward | 92% | 83% | 72% |
| % of landings in which the bird’s first foot adjustment was backward | 4% | 9% | 8% |
| % of landings in which the bird’s first foot adjustment was to the side | 4% | 8% | 20% |

**Supplementary file 1B. Individual foot preferences and adjustments during landing.**

| Perch | Parrotlet 1 | Parrotlet 2 | Parrotlet 3 | Averages by Surface |
| --- | --- | --- | --- | --- |
| Coast Live Oak | 1.67 | 3.00 | 2.67 | 2.44 |
| Floss Silk | 1.67 | 2.00 | 1.67 | 1.78 |
| Sweet Olive | 2.33 | 2.00 | 1.67 | 2.00 |
| 1.5” Diameter Birch Dowel | 1.67 | 1.67 | 2.00 | 1.78 |
| 0.75” Diameter Birch Dowel | 2.67 | 1.67 | 1.67 | 2.00 |
| 0.25” Diameter Birch Dowel | 2.33 | 1.00 | 0.67 | 1.33 |
| Foam | 2.00 | -- | 1.67 | 1.83 |
| Teflon | 3.67 | 1.67 | 3.00 | 2.78 |
| Sandpaper | 2.33 | 1.67 | 2.33 | 2.11 |
| Averages by Individual | 2.26 | 1.83 | 1.93 | 2.01 |

**Supplementary file 1C. Parrotlet foot adjustments on each perch.**

|  | Mean | Standard Deviation |
| --- | --- | --- |
| Resting Foot Spread Angle (˚) | 35.87 | 18.31 |
| Time from First Foot Movement to Contact (ms) | 91.86 | 14.64 |
| Peak Foot Angle (˚) | 171.57 | 10.33 |
| Peak Foot Angle Time Before Contact (ms) | 31.23 | 9.57 |
| Change in Foot Angle During Preshaping (˚) | 23.88 | 10.06 |
| Pre-Contact Claw Angle (˚) | 177.16 | 7.06 |
| Change in Foot Angle During Wrapping (˚) | 29.72 | 22.27 |
| Change in Claw Angle During Wrapping (˚) | 41.51 | 19.27 |
| Time of Maximum Claw Curl (ms) | 84.64 | 80.04 |

**Supplementary file 1D.** **Foot kinematics parameters**.
